# With Our Editors

**Published:** 1906-09-15

**Authors:** 


### WITH OUR EDITORS.

**THE DENTIST'S RANK.** *Note.—We publish the following not to endorse its sentiment, but because as its author entitled it, it cantains "A Few Gentle Thumps," and may help us to avoid being the kind of a citizen it portrays.*

Dentists oftentimes complain that they are not rated high in the community in which they live. They feel that the public does not properly recognize that they belong to a learned profession,and especially do some complain that the dentist is not regarded equally with the physician. The truth is that dentists as a class do not deserved to be ranked high in a community, and that they get just exactly the recognition they deserve and no more. If any dentist gets puffed up with his own importance and imagines that either he or his profession is equal to the physician and the medical profession, then Mr. Dentist richly deserves a few good bumps for becoming so chesty.

The truth is that dentistry, as followed by nine-tenths of its practitioners, is a narrow and a narrowing profession. The dentist first of all shuts himself up in an office, and often spends his whole day in a little cell, by courtesy called an operating room. Here he sees and comes in contact with what happens to come to him. He is not led by his work into the highways and byways of the world, but like the oyster, stays in one place and picks up what comes his way that is to his liking.

He does not see the seamy side of life like the physician, is not called to hovel as well as to palace, is not taken hither and thither from those that mourn because of the hand of death to those that rejoice over the coming of new life. The dentist is not called to the home, the hospital, the mine, the railroad wreck, the autopsy, and is not required to be physician, surgeon pharmacist, nurse, health officer, priest, counselor, and friend all in one. The dentist has his hours makes his appointments does his work, charges a little better fee than he thinks the other fellow gets, and smiles the smooth, oily smile of self-satisfaction.

The dentist does not enjoy the advantages of the lawyer, in being compelled to look into all kinds of questions, to study public affairs, or to hold his own in the intellectual battles he wages before the court or elsewhere, to meet keen wits and nimble tongues with citations, precedents and rulings that require a broad knowledge of the world's affairs. The dentist seldom has more weighty problems than the size and shape of a hole in a tooth, the texture and color of a piece of porcelain, the manipulating of gold foil, plate, or solder, and the fees involved do not often amount to more than one hundred dollars.

The ordinary dentist has no special claim to deep learning. He is not required to be even a high school graduate to start, and he goes through most of this studies with the idea to get through rather then to really master them. While he studies anatomy, physiology, chemistry, material medica, therapeutics, histology, pathology, bacteriology and embryology, he never has the same incentive to study these or takes the same interest in them as the medical student. The medic knows that these are the foundations of his profession and his later success, while the dental student regards them as side issues—"sometimes handy to know, but not much use." The course in these studies is usually less complete than the medical course in the same, and lacking the incentive to study and having an inferior course, the dentist can truthfully claim but the smattering of the branches.

Most dentist are deficient in general scientific knowledge. Few have ever studied much in physics or electricity, and volts, ohms, amperes, induction, kilowatts and resistance are very much like Choctaw. They are usually deficient in higher mathematics, and a mixed equation is as Greek to a bulldog. Neither have they studied the natural sciences related to physiology, anatomy, or chemistry, namely, zoology, botany, geology, or morphology. Few can read German or French, and thus miss more than half of modern science. What the ordinary dental student wants to learn is "to fill teeth and make money," and to get this knowledge in the quickest and easiest way. Science has little attraction compared with this shining gold.

Dentists very seldom mix in public affairs, not even performing the duties belonging to good citizens, for fear they may antagonize some patient and lose a dollar. For this reason they shirk their political and civic duties, and are never a positive help to the better element in a fight for good government, honest administration, public economy, clean streets, parks and drives, good schools and all the many public affairs of vital interest to all. A few dentists have proved exceptions to this tendency and have been made mayors and other public officers, but most of the profession would get cold feet at once if mentioned for the office of city councilman, park commissioner or school trustee. They are even somewhat doubtful as officers in the Church or Sunday-school.

It is undeniable that the dentist is not much as a business man. Most of the profession are careless about both collecting and paying bills. They usually make money, but seldom save any. Many are prudent enough to secure them homes, and some few accumulate a competence from their profession and other business enterprises. But the large majority spend their money as fast or faster than they make it, and die poor—sometimes in absolute want. The city dentists—the ones with the biggest practices—are the most imprudent in this respect.

The daily round of the average dentist is something like this: He arises, dresses, and takes the morning meal, then at once starts for the office, where he is soon busy in the work of the day. A short stop is made at noon for lunch, and then the office again. Usually the office claims all the hours fit for any outdoor exercise, and it shuts the dentist in from the great world about him, and away from the human interest on all sides. No wonder the public does not rate him high. It knows nothing about him, shut up in the everlasting office.

The dentist's occupation does not naturally bring him to the public eye, he does nothing himself to draw attention. He shirks all kinds of public duties, he does not figure as a business man, he cuts no ice as a capitalist or investor, he does not rank as a scientific man, and he is too shut up in his office to even be much of a sociable creature. We are not "knocking" when we say these things, but are aiming to present things just as they are, and we have no patience with the complaint that the public does not rate the dentist as he should be. On the other hand we maintain that the public rates the dentist just where he belongs, and that there is no cause for appeal from the public's verdict.

The dentist is just a respectable, useful, necessary member of a community, neither insignificant nor prominent. He lives a retired and busy life, minds his own business, and is neither hilariously welcomed when he arrives nor seriously missed when he dies. He averages up well with the ordinary run of humanity, and there is no cause to mourn over this standing or his behavior save when he forgets himself and imagines himself bigger than he really is.—Dr. W. J. Brady, in July *Western Journal.*

**DENTISTRY FOR PUBLIC SCHOOL CHILDREN IN GERMANY.**

That decay of the teeth is an active factoi in general physical degeneracy; that sound teeth are essential to sound digestion, and their diseases the cause, both direct and indirect, of many systemic disorders leading to individual and racial deterioration are axiomatic truths now generally recognized. Equally well established is the fact that in a great majority of instances the initiatory stages of dental lesions are observable in early childhood, and that if not then checked progressive pathological results inevitably follow. For these reasons it is fully apparent that the only sure method or arresting dental disease is to begin with its early manifestations. Notwithstanding the advances in dentistry it is obvious that this result has not been attained in any adequate degree, for the children of the masses of the people, and their dental inspection when assembled in public schools seems to be the only expedient offering much hope of securing better results.

In many American cities the general medical inspection of public school children is now operative and usually is inclusive of the oral cavity. It is, however, evident that such an inspection conducted solely by medical men must be superficial and inadequate. Furthermore, the discovery of dental disease is of but little advantage if not followed by appropriate treatment, and this for the children of the very poor must be gratuitous.

While in America dentistry for public school children has been much talked about, in Germany two cities have had such a system in successful operation for nearly four years. The movement had its beginning in Strassburg, where a school dental clinic was opened October 15, 1902. This was followed by Darmstadt, in which city, in December 1902, a commodious suite of rooms devoted to clinical uses was opened with public ceremonies in which city and State officials and leading resident dentists and physicians participated.

So much interest in these school dental clinics has been manifested by the dental profession and the educational authorities of other European countries, as well as of America that O. Koeller, Dental Surgeon in Charge of the Darmstadt Clinic, and Prof. Dr. Ernest Jessen, Director of the School Dental Clinic in Strassburg, have published, jointly, a pamphlet descriptive of the operation of their respective clinics from their foundation up to April 1906 (Die Zahnartzliche Behandlung der Volksschulkinder der Haupt und Residenzstadt Darmstadt und die Stadtische Schulzahnklinik in Strassburg im Elsass.)

An interesting feature of this pamphlet is a diagram showing the arrangement of the clinic rooms in Darmstadt and photographic views of their interior, the rooms, six in number, consist of two operating rooms, a waiting room, a toilet room, a consultation room for the officers of the clinic and a combined library and laboratory. As shown by the photographs these rooms are all surprisingly neat an attractive in appearance, with curtained windows, decorated walls, rugs for the floors, etc., while the equipment of dental apparatus and the other appointments, such as dental chairs, engines, glass tables and cabinets, are all of the most modern type. In general details nothing better is needed for any private dental office. The Strassburg clinic appears to be less elaborately housed, the rooms and their appointments being more upon the usual school-house lines, but the equipment has all the essentials for satisfactory dental work.

When first opened, clinical service was given by local practitioners without remuneration, but with the progress of time the work grew to such proportions that the employment of paid assistants was found necessary.

Concerning the results of the clinic in Strassburg, Consul Joseph I. Brittain has furnished the following report to the State Department at Washington.

"At the Strassburg clinic 5,333 children were examined the first year, and 2,666 received treatment. During the second year 6,900 were examined, and 4,967 were treated. The third annual report, just published, states there were 12691 visits to the clinic in 1904, and 6,828 children were treated, for whom 7,065 teeth were filled and 7,985 were extracted, and 4,372 other children had their teeth examined.

"Great advancement was made during the past year in the atten tion given children between the ages of three and six years. Of the children of these ages examined only 362 out of 2,269 had sound teeth, or less than 16 percent. Of the children examined between the ages of six and eight years, 160 out of a total of 2,103 had sound teeth, or but 7 1-5 percent. The school teacher enters the name of each pupil on a card, which is taken to the clinic, where the dentist enters a detailed record of the condition of the teeth after making the examination, returnng the card to the pupil and retaining a duplicate of the same.

"There were forty-four examination days, at which eighty children were examined per hour. The children were taught to clean their teeth three times daily, and especially before retiring. The dentist also instructs the children in the use of the tooth brush, each child receiving a brush for home use. The dentist also gives each child a piece of rye bread, and instructs him how to masticate the same with the least injury to the teeth.

"At a recent meeting of the Strassburg Teachers' Fraternity, where 400 teachers were present, a lecture was given on the observation and care which teachers should take in reference to the teeth of their pupils. At the meeting a practical demonstration was given by a class of boys between the ages of ten and twelve years, who showed remarkable knowledge concerning the instruction, diseases and care of the teeth. Since the introduction of the treatment there is a marked improvement in the general hea.th of the public school children, and there is less headache, earache and stomach trouble.

This unprejudiced report from a representative of the United States Government, if widely disseminated could not fail of effect in influencing public opinion in this country. It is evident that before the dental inspection of public school children can be instituted here a vast amount of indifference and skepticism must be overcome. First of all, the public must be convinced that the object of such a movement is the public welfare and not primarily and chiefly the personal profit of dentists.

In the report of the Committee on Oral Hygienic and Public School Dental Education of the Pennsylvania State Dental Society, made at the recent meeting in Philadelphia, the Society was urged to "endorse and encourage the introduction in public schools of periodic lectures on the subject of the teeth by competent practitioners." This is an excellent suggestion. To arouse the interest of children and of their teachers on the subject will go far toward securing the co-operation of parents in the movement to secure dental inpection for public schools.

An even more effectve aid to such a propaganda would be a pamphlet descriptive of what has been accomplished in Germany, illustrated with views of the Darmstadt clinic rooms and accompanied by a concise exposition of the facts which make the dental inspection of public school children desirable and necessary. If such a pamphlet were prepared under the auspices of the above named Committee, the Pennsylvania State Dental Society could hardly make a wiser disposition of a portion of its surplus funds than as an appropriation for its publication and distribution to boards of education, school directors and teachers throughout the the state.—Dr. W. F. Litch, in August *Brief*.

**ADVANCEMENT AND CONSERVATION OF ENERGY.** There are many ways to keep up with the profession, and also to keep up with the procession, and one of them is by means of your dental journal. I think that every dentist ought to take one or two good journals devoted to his profession. Another means of advancing in the profession, is to keep buying new text-books, and that is a point where most of us fall down. The rapid advances the profession has made in the last three decades render the old text-books almost obsolete in a very few years, in many particulars. I do not think there is any text-book on operative, prosthetic or other branch of dental art that is authority today, which was published six years ago,unless it has been revised; and if we wish to keep up with these rapid advances of our profession, we must cultivate the habit of buying the new text-books, as they come out.

A third valuable method of keeping up with the times, is by means of the dental society. Now everything that is new, and everything that is of value in the art and science of dentistry are brought to your attention in the Dental Society. It may have been brought to your attention in other states and in other ways first, but it comes to you before long in your dental meetings, and some member will write a paper on it, or it may be brought up in the discussion and thus you are brought more intimately in touch with these valuable things and can put them into practical use.

In addition to the three things I have enumerated I want to say a word or two on the subject "The Conservation of Energy." Energy can be conserved in the dental office, and it ought to be conserved. You ought to have all the labor-saving appliances that you can have. The dentist has only a limited number of years in which he can do his work, as compared with the members of other professions. The older a physician gets, if his mind is clear and his faculties unimpaired, the more he improves and he has the greater capacity for serving his clientele, because of his increased experience. That is not so true of the dentist in general practice, and so much more is it necessary that he should have every aid in the world to conserve his energy.

If a dentist finds that his practice is growing so that he can not attend to his work without going to his office at unusually early hours, or remaining at work until the chickens have gone to roost, he had better raise his prices immediately. That is a sure cure for overwork in the dental office. He will not have to go to his office at five o'clock in the morning, if he will charge more for his time, and if he will so increase his fees, that he receives as much compensation for working six hours, as he formerly did for working twelve, he is at least six hours to the good, and has that much time for his own particular use. That is one of the ordinary methods of conserving energy.

Another excellent method of conservation of energy, is to forget the shop when you leave your office. The writer of the paper spoke of several means by which energy can be conserved out of the office in other lines of life. They are all good, and they can be multiplied. He mentioned photography, and I dabble in that to the extent of ''pressing the button,'' which I did several times at the Kansas City Country Club. Golf is a good way to conserve your energy; tennis and many other atheltic sports are most excellent in their way. If you have any musical talent, that affords you a good way to employ the mind upon things outside of your daily occupation. If you have a talent for dramatics, if you can exercise that, and also keep away from the missiles that might from time to time come your way, it will afford a pleasant relaxation. Reflection outside of the office is a conservation of energy. You can no more expand a man's mentality, by a continual thinking in one groove, than you can expand all the muscles of the body by using one muscle. You have to exercise all sides of your mentality, and your brain power, to develop it as it should be developed. So, when you get out of your offices, dismiss your office cares. Devote such time to your office as is necessary to fulfill your duty to your patients to the fullest extent, to get the most out of your practice that you can possibly in benefit to your clientele, and when you leave your office, unless you are going to a dental meeting, then conserve your energy,—forget dentistry and forget your office cares and perplexities.

G. E. HUNT—*Western Journal.*

